# Utilization of a Multi-Tissue Extracellular Matrix in Complex Wound Care in Gaza: A Case Series

**DOI:** 10.3390/antibiotics14090885

**Published:** 2025-09-02

**Authors:** Bilal Irfan, Adam Hamawy, Ruba Musallam, Rahaf Abudagga, Sameer Khan, Nour Alshaer, Mohammed Tabash, Abdullah Ghali, Khaled Saleh, Mohammed Tahir

**Affiliations:** 1 Center for Bioethics, Harvard Medical School, Boston, MA 02115, USA; birfan@umich.edu; 2 Center for Surgery and Public Health, Brigham and Women’s Hospital, Boston, MA 02120, USA; 3 Department of Neurology, University of Michigan Medical School, Ann Arbor, MI 48109, USA; 4 Department of Epidemiology, University of Michigan School of Public Health, Ann Arbor, MI 48109, USA; 5 FAJR Scientific, Houston, TX 77041, USA; 6 Penn Medicine Princeton Medical Center, Plainsboro, NJ 08536, USA; 7 European Gaza Hospital, Khan Yunis, Gaza, Palestine; 8 Al-Aqsa Martyrs’ Hospital, Deir Al-Balah, Gaza, Palestine; 9 Faculty of Medicine, Islamic University of Gaza, Gaza P.O. Box 108, Gaza, Palestine; 10 Department of Orthopedics, Baylor College of Medicine, Houston, TX 77030, USA; 11 College of Human Medicine, Michigan State University, East Lansing, MI 48824, USA; 12 College of Medicine, Central Michigan University, Mount Pleasant, MI 48859, USA; 13 School of Medicine, Wayne State University, Detroit, MI 48202, USA; 14 Bart’s Health, London E1 2ES, UK

**Keywords:** stem cell, wound care, Gaza, trauma, conflict zones

## Abstract

**Purpose:** This case series examines the feasibility and outcomes of using a multi-tissue extracellular matrix (ECM) powder as an adjunct to standard wound care in a conflict zone. Primary objectives were granulation by day 7, wound closure, and minimizing early complications among patients with complex ballistic and blast injuries in Gaza during the 2024 Israeli military offensive. **Methods:** A retrospective observational study was conducted at the European Gaza Hospital from April to June 2024. Fifteen patients with high-energy soft tissue injuries who received ECM powder (XCellistem™) after surgical debridement were included. Data were extracted from operative reports, wound documentation, and clinical follow-up. Outcomes included granulation by day 7, wound closure method, and complications such as infection or dehiscence. **Results:** All 15 patients (median age 28; 14 male) sustained severe trauma, with 80% having exposed bone or tendon. ECM was applied directly to wound beds and often co-applied with vancomycin. Granulation tissue was observed in 12 patients by day 7, and 13 achieved wound closure via grafting, flap coverage, or secondary intention. No adverse reactions to ECM were reported. **Conclusions:** Multi-tissue ECM powder seems feasible and safe under austere conditions and appeared to support wound healing in severely injured patients. Its shelf stability, ease of use, and regenerative potential make it a promising adjunct for surgical care in resource-constrained conflict zones.

## 1. Introduction

Wound management in austere combat environments presents a unique set of challenges, particularly given a climate of resource limitations, a high influx of patients, and disruptions to supply chain and personnel availability amid armed conflict [1,2]. Traditional wound care modalities, although well-established in higher-resource settings, often prove difficult to implement effectively in conflict zones [3,4,5,6,7]. Over the last two decades, extracellular matrix powder-based (ECM) biomaterials have gained attention for their potential role in facilitating wound healing, particularly through supporting granulation tissue formation, modulating inflammation, and reducing infection and fibrosis [8,9,10,11,12,13,14].

Multi-tissue ECM products, which combine structural proteins (e.g., collagen, elastin, fibronectin) with glycosaminoglycans and bioactive factors (e.g., VEGF, EGF, FGF), offer a complex signaling environment intended to promote constructive tissue remodeling [15,16]. Such scaffolds have shown the potential to modulate the host’s inflammatory immune response from a predominantly pro-fibrotic and scarring (M1) phenotype to a more constructive, regenerative (M2) macrophage phenotype [17]. ECM products have been noted to decrease infection rates, bolster tissue regeneration, and potentially lower seroma formation when used in high-risk surgical wounds [18,19,20,21]. ECMs and similar products have in the past demonstrated favorable outcomes for healing in various types of wounds ranging from those with infection, certain types of cysts and ulcers, military-related wounds, or even complex trauma-related injuries [22,23,24,25,26,27,28,29,30,31,32,33,34,35,36].

The prevailing hypothesis is that in situations of severe soft tissue damage, whether from gunshots, explosive injuries, or large degloving wounds, adding a robust ECM scaffold can promote local tissue repair through chemotactic recruitment of progenitor cells, a more favorable macrophage phenotype, and improved angiogenesis [37]. While negative pressure wound therapy (NPWT) has demonstrated efficacy in managing these wounds, its dependence on electricity and consumables limits its utility in many low-resource or unstable environments [38,39,40,41,42,43,44,45]. In such settings, dry, shelf-stable ECM powders may provide an alternative or adjunct therapy, offering ease of application in both operative and bedside settings without requiring specialized equipment [46].

During the 2023–2025 Israeli military assault on Gaza, international medical teams faced a high burden of traumatic injuries among a displaced and malnourished civilian population [2,47,48,49,50,51,52,53,54]. In this context, a multi-tissue ECM powder was used as an adjunct to standard debridement and wound care in patients with complex ballistic and blast injuries. This case series documents the outcomes of 15 such patients treated in Gaza between April and June 2024. Our aim was to assess the feasibility of ECM biomaterials under conditions of extreme resource scarcity and malnutrition, with attention to granulation tissue formation, wound closure, and early complications such as infection or dehiscence.

## 2. Results

Fifteen patients (14 male, 1 female; age range: 9–67 years, median age: 28 years) with complex ballistic or blast-related extremity and soft tissue injuries were treated between April and June 2024. All patients underwent operative debridement, and each received at least one application of a multi-tissue extracellular matrix powder as part of their wound management protocol. Injuries included open fractures with exposed hardware, tendon or nerve transections, traumatic amputations, and infected soft tissue wounds in malnourished patients. Across the cohort, there were no documented adverse reactions directly attributed to ECM powder. A summary of wound outcomes is demonstrated in Table 1. Most wounds progressed to a clean, granulating bed suitable for delayed closure, skin grafting, or flap coverage within 1–3 weeks of ECM application.

To illustrate the spectrum of injury patterns and responses to treatment, seven representative cases are summarized below.

### 2.1. Case A (Upper Extremity Reconstruction)

A 42-year-old male presented with a comminuted fracture of the left thumb metacarpal with segmental bone loss and a digital nerve injury. He underwent K-wire fixation, flexor pollicis longus and nerve repair, and bone grafting. After thorough debridement, ECM powder was applied over the wound bed along with vancomycin. The wound was closed primarily. Follow-up at day 4 and day 10 showed good perfusion, minimal edema, and healthy granulation without signs of infection.

### 2.2. Case B (Pediatric Multi-System Injury)

**Figure 1:**
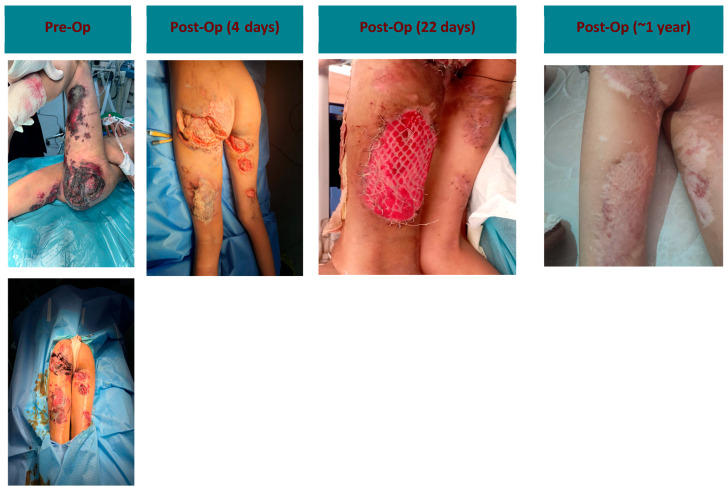
Gluteal region injury progression: pre-op to 1 year post-op.

A 10-year-old female suffered injuries from an explosive device, including bilateral lung contusions, liver laceration, a left pelvic fracture, and multiple deep extensive soft tissue wounds to the lower extremities and gluteal region. She underwent staged debridement and orthopedic fixation (Figure 1). ECM powder and vancomycin were applied to the raw areas. Granulation tissue developed within 10–14 days, enabling successful split-thickness skin grafting over the left thigh and gluteal region.

### 2.3. Case C (Traumatic Amputation and Limb Salvage)

A 14-year-old male with traumatic amputations of the left hand and right leg, along with open fractures and soft tissue injuries to both upper limbs, underwent multiple staged debridements, amputations, and reconstructions. ECM powder was used on the raw areas of both upper limbs. The wound beds treated with ECM powder rapidly granulated after washout. Subsequent skin grafts achieved near-complete take, with stable wound coverage at follow-up.

### 2.4. Case D (Complex Foot Injury with Exposed Bone and Tendon)

A 23-year-old male with a blast injury to the right foot had exposed tendon and bone following debridement of necrotic tissue. He underwent reduction in a Lisfranc dislocation with K-wire fixation and serial NPWT changes. ECM powder was applied during dressing changes. By day 30, the wound had developed robust granulation tissue, allowing for partial closure and re-epithelialization.

### 2.5. Case E (Infected Lower Limb Wound with Exposed Implant)

A 36-year-old male with an open tibial fracture underwent external fixation, later converted to intramedullary nailing. A medial soleus flap and skin graft were attempted, but the graft failed, leaving the nail exposed. ECM powder and NPWT were applied. Over the next two weeks, granulation tissue formed sufficiently to allow re-grafting, which achieved full take without further complication.

### 2.6. Case F (Explosive Thumb Injury with Skeletal Fixation)

**Figure 2:**
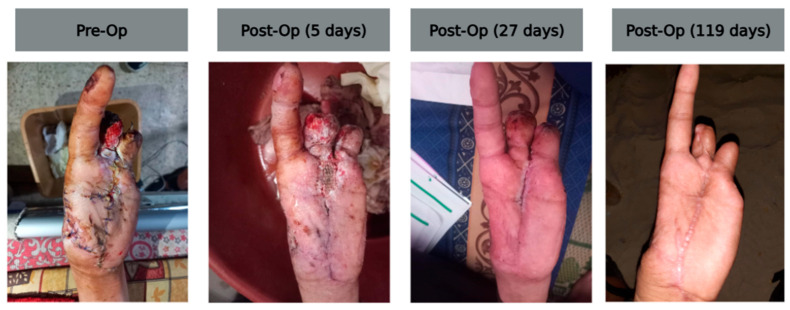
Hand injury progression: pre-op to 119 days post-op.

A 23-year-old male presented with thumb gangrene and soft tissue loss following an explosive injury, which was complicated by prior misaligned wiring after initial debridement. On 5 June 2024, he underwent removal of necrotic tissue and old sutures, followed by re-debridement. ECM powder and topical vancomycin were applied to the wound bed, and two cross K-wires were placed through the metacarpal head for stabilization. The wound demonstrated progressive granulation and epithelialization and achieved stable closure (Figure 2).

### 2.7. Case G (Complex Upper Arm Injury with Nerve and Vascular Repair)

**Figure 3:**
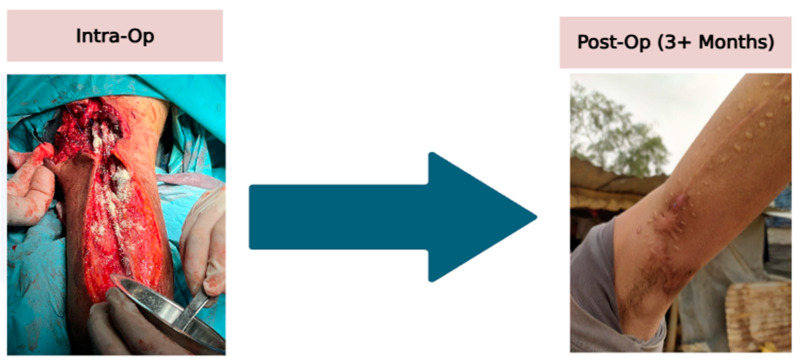
Intra-operation to post-operative arm with ECM powder application.

A 17-year-old male sustained a large medial upper arm wound from an explosive device. Intraoperative exploration on 5 September 2024 revealed a 3 cm segmental loss involving approximately 90% of both the median and ulnar nerves, with the radial nerve intact, along with brachial artery contusion and thrombosis. Four fascicular nerve grafts were performed on each injured nerve, targeting the medial cutaneous and medial cord fascicles, and thrombectomy of the brachial artery was completed. ECM was applied before primary closure. The wound healed without dehiscence (Figure 3).

## 3. Discussion

During the Israeli military assault on Gaza, international healthcare teams operated under conditions of extreme scarcity, treating large volumes of trauma patients amidst infrastructure collapse, population displacement, and widespread malnutrition [2,55,56,57,58,59,60,61,62,63]. In this context, the use of biologically active wound care materials offered a potential adjunct to traditional debridement and delayed closure strategies. One such material, XCellistem™, a multi-tissue extracellular matrix powder, was utilized for its ease of application and potential to promote granulation in complex or contaminated wounds.

XCellistem™ is derived from multiple porcine organs, including spleen and lung, and is processed into a dry formulation that can be applied directly to irregular wound beds, including those with exposed bone or hardware [15]. Unlike single-source collagen products, this multi-tissue matrix contains a diverse array of bioactive molecules such as various collagen subtypes, elastin, fibronectin, laminin, proteoglycans, glycosaminoglycans (GAGs), and growth factors including fibroblast growth factor (FGF), vascular endothelial growth factor (VEGF), and epidermal growth factor (EGF) [15]. These components are believed to support constructive tissue remodeling by modulating the wound microenvironment, encouraging angiogenesis, and facilitating the recruitment of endogenous repair cells [3,16]. Materials that modulate the immune response toward constructive processes may help achieve a more stable and functional repair [64,65]. Preclinical studies have suggested that such ECM materials may also shift the immune response toward an M2 macrophage phenotype, associated with regenerative rather than fibrotic healing [66]. Histological analysis of wounds treated with XCelliStem™ demonstrated a favorable tissue remodeling response characterized by collagen deposition, neovascularization, fibroblast and macrophage infiltration, and also an absence of foreign-body granulomas, showcasing its biocompatibility and immunological safety [67]. Furthermore, clinical outcomes reported in prior case series have noted that treatment with XCelliStem™ facilitated rapid wound closure without re-infection or reoperation, thus potentially reducing costs compared to traditional negative pressure wound therapy [67].

In Gaza, where standard wound care practices were constrained by power outages, post-surgical infection rates are high, supplies are limited, and there is inconsistent access to sterile equipment or negative-pressure systems, the ECM powder’s dry, shelf-stable formulation allowed for flexible use in the operating theater or at bedside [68,69,70,71,72,73,74,75,76]. Its application required no specialized devices and could be combined with topical antimicrobials such as vancomycin powder in wounds at high risk for infection. The case series presented here illustrates how this material was incorporated into the care of patients with high-energy soft tissue injuries, often following ballistic or explosive trauma. ECM powder was applied in this case series as a structural and immunomodulatory scaffold rather than as an antibacterial agent, and further research would be needed to clarify any intrinsic antimicrobial activity. Nevertheless, prior work suggests some ECM scaffolds can indirectly limit bacterial colonization via sequestration of microbes and release of endogenous peptides, although effects may be material-specific and not generalizable [77].

Data from the World Health Organization’s (WHO) Eastern Mediterranean region shows that conflict-affected settings carry among the heaviest burden of antimicrobial resistance [78,79]. In Palestine, antimicrobial resistance (AMR) rates are quite high; ESBL production is reported in 27% of *E. coli* and 40% of *K. pneumoniae isolates*, and Gaza hospital tap/wastewater has contained OXA-producing *Enterobacteriaceae* with substantial imipenem resistance; clinicians have also documented large swaths of AMR infection among gunshot-wound patients during the 2018 Great March of Return [80]. While there were substantial concerns regarding potential antibiotic resistance given the setting of armed conflict, the manufactured unavailability of the most basic medical supplies and drug shortages necessitated the use of vancomycin [2,81,82].

The findings from this observational series indicate that multi-tissue ECMs may offer a useful adjunct in the treatment of complex high-energy wounds in resource-constrained settings. Clinicians observed progressive granulation tissue formation in wounds treated with ECM powder following standard debridement and dressing protocols, even in the presence of exposed bone, devitalized soft tissue, and external fixation hardware. Given the austere conditions, limited power, staff shortages, and lack of advanced wound care systems, the dry, shelf-stable nature of the ECM dressing provided practical advantages. In many cases, ECM powder was applied in conjunction with local antibiotic powders such as vancomycin, offering both a structural scaffold and infection control within the same operative field. This combination appeared to support wound bed progression, potentially mitigating some of the infection risks well-documented in surgical interventions in Gaza [68].

While this series lacks a control group or randomization, clinical observations were consistent across cases: wounds treated with ECM powder often exhibited early signs of granulation, leading to grafting or secondary closure within a relatively short time frame (Figure 2) [83]. The product’s multi-tissue composition includes a mixture of collagen types, elastin, fibronectin, laminin, glycosaminoglycans, and growth factors [8,84]. These offer a broader biological signaling environment than traditional collagen-only dressings. This complexity may enhance recruitment of macrophages, fibroblasts, and endothelial progenitor cells involved in vessel formation and matrix remodeling [8,12]. In contaminated wounds, particularly those sustained in combat settings, the ability to shift the inflammatory response from a destructive to a constructive trajectory is a clinically relevant feature [85,86,87]. In this series, ECM was applied as an adjunct or alternative to NPWT or wet-to-dry dressings, and direct comparisons were not feasible here; future studies comparing ECM-first versus NPWT-first algorithms in resource-limited settings may be of interest.

It is important to note, however, that multiple confounding factors limit interpretation. Surgical decision-making varied across cases depending on available personnel, timing of presentation, and fluctuating access to NPWT systems. Some patients received single applications of the ECM product, while others underwent repeated use prior to closure or grafting. Additional heterogeneity existed in closure techniques, ranging from delayed primary approximation and local flap coverage to healing by secondary intention. Furthermore, no standardized laboratory markers or serial imaging were available to corroborate clinical assessments. Each wound’s unique characteristics, including extent of contamination, vascular compromise, or presence of necrotic bone, may have independently influenced healing outcomes.

Furthermore, this descriptive case series lacks a contemporaneous non-ECM comparator; therefore, we cannot attribute outcomes to ECM powder independent of thorough debridement, antibiotic usage, or natural healing processes. Findings should be interpreted as associations observed under extreme constraints rather than definitive estimates of treatment effect. Similarly, the heterogeneity in age, mechanism, anatomic site, contamination, and vascular status, combined with variable access to fixation and NPWT, limits the generalizability of these findings that emerge from a cohort of 15 patients. This variability likely confounds time-to-granulation and closure and precludes more formal statistical assessments of significance.

Despite these limitations, no adverse effects specific to the ECM product were observed, and surgeons repeatedly reported favorable tissue response, even after repeat debridements. Failures of graft take, where present, were most often associated with infection or inadequate vascularity rather than with the scaffold itself. These results are consistent with prior data suggesting that well-processed ECM materials can avoid triggering foreign body reactions and instead foster a reparative immune phenotype [66].

From a logistical standpoint, ECM powders may offer advantages over more complex wound systems in conflict settings. Negative pressure wound therapy remains a valuable tool in soft tissue reconstruction but is frequently hampered by device shortages, its reliance on electrical supply, disposable canisters, and trained staff. In contrast, ECM materials require minimal resources and can be stored, transported, and applied with relative ease. In Gaza, many patients were managed in overstretched local clinics between operations, and the ability to reduce dressing change frequency and maintain wound stability over time was critical.

Prospective studies and standardized documentation, although difficult to conduct in war zones, would help clarify the specific benefits of ECM use versus standard care or other biologic dressings [88,89,90,91,92]. Ideal endpoints would include time to definitive closure, infection rates, resource utilization, and cost-effectiveness. Immunohistochemical studies of wound biopsies could offer further insights into mechanisms of action, particularly shifts in macrophage phenotype, vascular ingrowth, and matrix remodeling. Such research could guide future material development and inform clinical triage strategies in crisis settings.

War-related wounds often involve large zones of tissue necrosis, heavy contamination, and unpredictable vascular damage from embedded fragments [93,94,95,96,97,98,99,100,101,102,103,104]. The ability to deliver a structurally and biologically active material into irregular wounds and maintain effectiveness despite contamination presents a valuable treatment option. Even without long-term functional data for this cohort, the movement toward stable closure in most cases is an important clinical milestone, especially in a population with limited access to rehabilitation or secondary reconstruction. In a besieged and blockaded territory where healthcare infrastructure is under systemic attack, achieving wound closure with minimal reoperation becomes a priority not only for individual recovery but also for preserving surgical capacity. ECM-based materials may help bridge this gap where conventional methods fall short.

## 4. Methods

### 4.1. Study Design and Setting

This is a retrospective observational case series performed in the European Gaza Hospital (EGH) in Khan Yunis, Gaza Strip, Palestine. The period of study was April 2024 to June 2024, coinciding with a surge in trauma cases during the Israeli military offensive in the southern governorate of Rafah.

During this time, the hospital was only partially operational. Widespread shortages of staff due to displacement, abduction, or death, combined with limited surgical and anesthetic equipment, frequent power outages, and an inconsistent supply of advanced wound care materials, posed significant barriers to care delivery [105]. Standard protocols for operative sterility were difficult to maintain. In addition, severe malnutrition among the patient population further impeded wound healing and recovery.

NPWT was employed when functional equipment and sponge dressings were available; however, it was frequently unavailable or unsustainable. In many cases, it was supplemented or replaced by simpler methods, including basic wet-to-dry dressings and the application of ECM powder (XCellistem™, RTT Medical, Orlando, FL, USA). XCelliStem™ is an FDA 510(k)-cleared wound dressing (K172593) utilized for the management of partial- and full-thickness wounds, pressure ulcers, diabetic and venous ulcers, surgical wounds, second-degree burns, and trauma wounds including abrasions, lacerations, and skin tears [106,107,108]. The device is supplied sterile for single topical use and consists of porcine-derived extracellular matrix sourced specifically from spleen and lung tissue.

### 4.2. Selection of Cases

A total of 15 patients (14 male, 1 female) who sustained high-energy ballistic or explosive injuries were included in this series. The inclusion criteria were as follows: (1) presence of complex wounds with soft tissue loss and/or bone exposure, (2) requirement for at least one operative debridement, and (3) an application of a multi-tissue ECM powder (XCelliStem™, RTT Medical, Orlando, FL, USA) at least once during the wound management period.

Patients were excluded if they received other advanced ECM products (e.g., porcine small intestinal submucosa) to avoid confounding or if they succumbed to their injuries prior to any formal wound evaluation being performed. For each patient, data were extracted from operative notes and included demographics (age, gender), date of injury, mechanism of injury, the nature of injuries, operative procedures, antibiotic administration, and the number and timing of XCellistem™ (hereafter referred to as ECM powder) applications.

### 4.3. Intervention Protocol

All patients underwent operative debridement to remove devitalized tissue, followed by irrigation with normal saline. When indicated, surgical stabilization of fractures was performed using external fixation, intramedullary nailing, or K-wires.

Following thorough debridement, multi-tissue extracellular matrix powder was applied directly to the wound bed, with particular attention to areas of exposed bone, tendon, or neurovascular structures. The amount used typically ranged from 0.5 to 1 g, depending on the size and complexity of the wound.

In many cases, vancomycin powder was co-applied to reduce bacterial load. Wounds were then covered with a non-adherent primary layer (e.g., petroleum gauze or contact layer) followed by a secondary absorbent dressing. Dressing changes were performed every 3 to 5 days, or more frequently if clinically indicated, in accordance with local practice patterns. Repeat applications of ECM powder were performed in some cases, based on wound progression and the judgment of the attending surgeon.

### 4.4. Outcome Measures

The primary outcomes assessed in the study included the following: (1) evidence of granulation tissue formation by postoperative day 7 or during subsequent dressing changes; (2) wound closure achieved through secondary intention, split-thickness skin graft (STSG), local flap coverage, or primary approximation when feasible; and (3) the occurrence of early complications such as persistent purulent discharge, deep infection, re-operation, or wound dehiscence. Granulation by day 7 was assessed clinically by the attending surgeon during scheduled dressing changes using standard bedside criteria, including the appearance of red granulation tissue with punctate capillary oozing and coverage of ≥50% of the wound bed. This was further corroborated by photographs whenever feasible. In this setting, utilizing standardized scoring systems or multiple-rounded blinded adjudication was not possible.

Data were retrospectively collected from operative notes, nursing wound care documentation, and photographic records when available. No formal laboratory markers or advanced imaging studies were utilized, apart from standard radiographs to assess fracture healing. Follow-up periods varied from 2 to 8 weeks, depending on patient discharge timing and availability for re-evaluation.

## 5. Conclusions

In the context of war-related trauma and overwhelming systemic collapse, the ability to achieve wound closure with limited resources is vital. This case series suggests that a multi-tissue extracellular matrix dressing, applied in conjunction with thorough debridement and local antimicrobial strategies, was both feasible and well-tolerated in a severely resource-constrained environment. While the absence of a control arm limits the strength of inference, the clinical outcomes observed in terms of granulation and readiness for closure suggest that ECM materials may serve as an effective adjunct to traditional wound care. Their ease of use, stability without refrigeration, and compatibility with contaminated or complex wounds make them especially suitable for austere surgical settings. Mechanistic biopsy sub-studies examining macrophage polarization and vascular ingrowth may help clarify which wounds derive the most benefit. Until such data are available, ECM powder may be considered a feasible adjunct when electricity, consumables, and staffing limit advanced wound-care systems. Future prospective studies are warranted to evaluate comparative effectiveness and long-term outcomes, but the results presented here underscore the potential role of biologic scaffolds in humanitarian surgical care.

## Figures and Tables

**Table 1:** Summary of wound characteristics among 15 patients treated with ECM powder.

Characteristics	Number of Patients (n = 15)
Mechanism of injury: Explosive trauma	10
Mechanism of injury: Gunshot wound	5
Exposed bone or tendon at time of ECM application	12
Required ≥ 2 surgical debridements	11
Received concurrent vancomycin powder	13
Evidence of granulation tissue by day 7	12
Wound closure via skin graft or flap	9
Wound closure via secondary intention	4
Adverse reaction attributable to ECM	0

## Data Availability

The data presented in this study are available on request from the corresponding author due to privacy concerns and an active armed conflict.
